# Novel MRI metrics: Is targeting brain atrophy a practical treatment goal? Commentary

**DOI:** 10.1177/13524585261433911

**Published:** 2026-03-29

**Authors:** Anna Karin Hedström

**Affiliations:** Department of Clinical Neuroscience, Karolinska Institutet, Stockholm, Sweden; Department of Neurology, Karolinska University Hospital, Stockholm, Sweden

Over the past two decades, MRI in multiple sclerosis (MS) has shifted from detecting focal inflammatory lesions to quantifying diffuse tissue loss. As attention has increasingly turned to mechanisms underlying long-term disability, brain atrophy has assumed a more prominent role in disease monitoring.

De Stefano and Bianchi argue that the rationale for incorporating brain volume loss (BVL) into treatment goals is already strong. BVL reflects structural damage that lesion activity does not fully capture and correlates with physical disability.^1^ Evidence linking BVL to progression independent of relapse activity strengthens the argument that controlling inflammatory lesions alone does not fully address disease activity.^2^ In randomized trials, treatment effects on atrophy parallel effects on disability, in some analyses partly independent of lesion suppression.^3^ In progressive disease, regional measures, particularly deep gray matter and thalamic atrophy, show consistent associations with clinical outcomes.^4^ These observations make a purely inflammation-focused monitoring strategy increasingly difficult to defend.

The reservations raised by Strijbis and Schoonheim concern implementation rather than relevance. Annual volume changes are small, and measurement variability across scanners and protocols can approach the magnitude of true biological change. Non-disease-related factors such as aging, hydration status, and comorbidities influence brain volume, and pseudoatrophy after treatment initiation further complicates longitudinal assessment.^1^ Most importantly, there are no widely accepted MS-specific longitudinal thresholds that define when observed volume loss should prompt treatment escalation.^5^ Without such standards, even a biologically sound metric cannot reliably guide individual decisions.

The divergence between these perspectives reflects different thresholds for action rather than disagreement about biological relevance. At the trial level, brain atrophy has established value as an outcome that captures aspects of progression beyond focal lesions.^3^ At the individual level, treatment decisions require stable imaging protocols, low measurement error, and validated age-adjusted reference data that can be applied across centers.^5^

There is also a distinction between global and regional metrics. Whole-brain atrophy provides a stable summary measure of tissue loss, whereas regional measures, such as thalamic volume, may offer greater biological specificity but are more demanding to quantify. Ongoing harmonization efforts and improved segmentation methods suggest that these technical constraints may be gradually decreasing, although implementation remains uneven across clinical settings.

Taken together, the evidence supports brain atrophy as a meaningful marker of disease progression and a valid endpoint in clinical trials. Its use as a direct treatment target in everyday clinical practice will depend on further standardization, validated longitudinal reference data, and integration into structured monitoring frameworks. At present, brain atrophy should inform, but not yet dictate, therapeutic strategy.
